# Measuring and characterizing night time human behaviour as it relates to residual malaria transmission in sub-Saharan Africa: a review of the published literature

**DOI:** 10.1186/s12936-019-2638-9

**Published:** 2019-01-11

**Authors:** April Monroe, Sarah Moore, Hannah Koenker, Matthew Lynch, Emily Ricotta

**Affiliations:** 1 PMI VectorWorks Project, Johns Hopkins Center for Communication Programs, Baltimore, MD USA; 2 Swiss Tropical and Public Health Institute, Basel, Switzerland; 3 University of Basel, Basel, Switzerland; 4 Ifakara Health Institute, Dar es Salaam, Tanzania

**Keywords:** Malaria, Residual transmission, Outdoor transmission, Review, Sub-Saharan Africa, Insecticide treated nets, Human behavior, Human-vector interaction, Human-vector contact

## Abstract

**Background:**

Malaria cases and deaths decreased dramatically in recent years, largely due to effective vector control interventions. Persistence of transmission after good coverage has been achieved with high-quality vector control interventions, namely insecticide-treated nets or indoor residual spraying, poses a significant challenge to malaria elimination efforts. To understand when and where remaining transmission is occurring, it is necessary to look at vector and human behaviour, and where they overlap. To date, a review of human behaviour related to residual malaria transmission has not been conducted.

**Methods:**

Studies were identified through PubMed and Google Scholar. Hand searches were conducted for all references cited in articles identified through the initial search. The review was limited to English language articles published between 2000 and 2017. Publications with primary data from a malaria endemic setting in sub-Saharan Africa and a description of night time human behaviours were included.

**Results:**

Twenty-six publications were identified that met inclusion criteria. Study results fit into two broad categories: *when* and *where* people are exposed to malaria vectors and *what* people are doing at night that may increase their contact with malaria vectors. Among studies that quantified human-vector interaction, a majority of exposure occurred indoors during sleeping hours for unprotected individuals, with some variation across time, contexts, and vector species. Common night time activities across settings included household chores and entertainment during evening hours, as well as livelihood and large-scale socio-cultural events that can last throughout the night. Shifting sleeping patterns associated with travel, visitors, illness, farming practices, and outdoor sleeping, which can impact exposure and use of prevention measures, were described in some locations.

**Conclusions:**

While the importance of understanding human-vector interaction is well-established, relatively few studies have included human behaviour when measuring exposure to malaria vectors. Broader application of a standardized approach to measuring human-vector interaction could provide critical information on exposure across settings and over time. In-depth understanding of night time activities that occur during times when malaria vectors are active and barriers to prevention practices in different contexts should also be considered. This information is essential for targeting existing interventions and development and deployment of appropriate complementary prevention tools.

## Background

Substantial and sustained global efforts have led to a significant decrease in malaria burden over the last 15 years, with a 41% decrease in incidence rates and 62% decrease in mortality [1, 2]. These efforts include large-scale distribution of insecticide-treated nets (ITNs), targeted indoor residual spraying (IRS), wider availability of affordable and effective artemisinin-based combination therapy (ACT), and intermittent preventive therapy during pregnancy (IPTp). An estimated 68% of the decrease in infections can be attributed to ITNs, making this the most effective malaria prevention tool currently available [1, 3]. Combined, the core vector control interventions, ITNs and IRS, account for an estimated three quarters of clinical malaria cases averted [1].

Despite the contribution of ITNs and IRS to vector control, malaria persists, with a disproportionate impact on sub-Saharan Africa. In 2016, sub-Saharan Africa accounted for 90% of all malaria cases and 91% of all malaria deaths [4]. Residual malaria transmission, defined by the World Health Organization as ‘*persistence of parasite transmission even with good access to and usage of ITNs or well*-*implemented IRS, as well as in situations where ITN use or IRS are not practical*’, represents a critical challenge for malaria control and elimination efforts [5–8].

As indoor-focused interventions, there are limitations to the protection ITNs and IRS can confer. This issue may be compounded by shifts in vector behaviour and species composition in response to vector control interventions across settings [6, 9–13]. Significant research has been done to understand mosquito feeding and resting behaviour. The dominant malaria vectors in Africa are *Anopheles gambiae* sensu lato (s.l.) (including *An. gambiae* sensu stricto, *Anopheles coluzzii* and *Anopheles arabiensis*) and *Anopheles funestus* sensu stricto (s.s.) [9, 14, 15]. Typically, *An. gambiae* s.s., *An. coluzzii* and *An. funestus* s.s. are anthropophagic and feed and rest indoors [4, 6], while *An. arabiensis*’ behaviour is more plastic, showing zoophagic and exophilic tendencies [16, 17]. The differences in biting and resting behaviours affect the success of interventions like IRS and ITNs, as mosquitoes that feed and rest inside are more likely to encounter insecticide than those who feed and rest outside. In addition, in recent years shifts in vector behaviour following introduction of malaria control interventions in certain locations have been observed [9–13, 18, 19]. These changes can include species shifts, shifts toward early evening and early morning biting, toward outdoor resting and biting, and toward zoophily [6].

While these observed shifts are a result of successful vector control, there is an urgent need to understand when and where people remain at risk for malaria transmission to effectively target specific places, groups, and activities. This information is critical for guiding malaria control and elimination efforts. To understand when and where remaining transmission is occurring, it is necessary to look at both vector and human behaviour, and specifically the times when they overlap. While significant attention has been given to vector behaviour, to date a comprehensive review of night time human behaviour has not been carried out. The aim of this review is to synthesize the current body of evidence on human behaviour as it relates to transmission that can occur in the context of high vector control coverage, and existing methods for measuring and characterizing this human behaviour. The review focuses on human behaviour in sub-Saharan Africa based on the disproportionate burden of malaria in these countries.

## Methods

A literature review of published research findings was carried out using electronic databases, specifically PubMed and Google Scholar. Search terms were developed and refined prior to beginning the review (Table 1). Articles were identified and screened if they included any combination of the search terms in the title, abstract, or the body of the article. Additional articles were identified through a hand search of all references in articles identified through the initial keyword search. The review was limited to English language articles published between January 1, 2000 and December 31, 2017.

**Table 1:** Search terms and resulting number of articles

Search terms Limits: species-human; publication dates: 01/01/2000-12/31/2017	Number of articles screened
Africa[MeSH Terms] AND human AND (behavior OR behaviour) AND malaria	1361
Africa[MeSH Terms] AND “Human Activities” [MeSH Terms] AND malaria	732
Outdoor OR outside OR residual AND malaria AND (behavior OR behaviour)	307
Malaria AND (outdoor OR residual) AND behavior	217
Human AND location AND malaria	119
(“Human behavior” or “human behaviour”) AND malaria	45
“Human activities”[Mesh] and malaria and (outdoor OR residual)	23
Anthropology OR anthropologic AND malaria exposure	17
Outdoor AND human AND behavior AND night AND Africa	21
Outdoor AND malaria AND (“human behaviour” OR “human behavior”)	6
Africa[MeSH Terms] AND “human exposure” AND malaria	35
Africa[MeSH Terms] AND “Human Activities” [MeSH Terms] AND night time	4
Africa[MeSH Terms] AND human AND (behavior OR behaviour) AND night time	12

Studies were included in the review if they involved investigation of human behaviours in relation to malaria exposure. Specifically, studies needed to include a malaria endemic setting in sub-Saharan Africa and a description of human behaviours occurring during times when malaria transmission can occur, i.e. when malaria transmitting vectors are active. Behaviour is defined by PubMed as, “the observable response of a man or animal to a situation,” and the term is used broadly in this review to encompass human activities, location, and sleeping patterns. This includes activities occurring within or nearby the home, within the community, or outside of the community. Abstracts for articles identified with the search terms were reviewed, and for those that met the above criteria, the full-text was evaluated and grouped by categories of human activities occurring during times when local malaria vectors are active, methods for capturing human behaviour, and presence and type of entomology data collected alongside the human behavioural data. Articles that included mosquito biting rates without measuring human behaviour and articles that described ITN use only were excluded.

## Results

A total of 26 articles were identified that met inclusion criteria. These studies provided information on two key areas of interest: when (time of night) and where (indoors versus outdoors) people are exposed to malaria vectors and characterization of night time activities occurring during hours when malaria vectors are active.

### Human exposure to malaria vectors

Ten studies integrated human behavioural and entomological data to provide a quantitative estimate of human-vector interaction occurring indoors and outdoors (Table 2).

**Table 2:** Studies quantifying human-vector interaction

Author (year) [reference]	Country	Human behaviour methods	Human behavioural information collected	Entomological methods	Timing of entomology and human behaviour data collection	Human exposure to malaria vectors
Killeen et al. (2006) [20]	Tanzania	Survey	Usual bed time and wake-up time	Indoor and outdoor HLC, 6:00 p.m.–6:00 a.m.	Mosquito collections: 1997 and 2004 Survey: 2002–2004	Indoor exposure for non-user: 90% Indoor exposure during sleeping hours (9:00 p.m.–5:00 a.m.): 80% Protective efficacy of an ITN: 70%
Geissbuhler et al. (2007) [21]	Tanzania	Survey and direct observation	Survey: Dinner location, location after dinner, usual bed time and wake-up time, use of prevention measures Direct observation: People outdoors for each hour of the night	Indoor and outdoor HLC, 6:00 p.m.–7:00 a.m.	Mosquito collections: April-June 2006 Survey: Carried out in same households; exact timing not specified	Indoor exposure for non-user (*An. gambiae* s.l.): 79% Indoor exposure during sleeping hours for non-user (*An. gambiae* s.l.): 74% Protective efficacy of an ITN (*An. gambiae* s.s.): 59% Protective efficacy of an ITN (*An. arabiensis*): 38%
Russell et al. (2011) [22]	Tanzania	Survey	Usual bed time Usual wake-up time	Indoor and outdoor HLC, 7:00 p.m.–7:00 a.m.	Mosquito collections: 1997, 2004, and 2009 Survey: 2002–2004	Indoor exposure for non-user (1997 *An. gambiae* s.l.): 92% Indoor exposure for non-user (2004 *An. gambiae* s.l.): 99% Indoor exposure for non-user: (2009 *An. gambiae* s.l.): 79% Indoor exposure for non-user (1997 *An. funestus*): 93% Indoor exposure for non-user (2004 *An. funestus*): 73% Indoor exposure for non-user (2009 *An. funestus*): 45%
Seyoum et al. (2012) [23]	Zambia	Survey	Usual time indoors for the night, bed time, wake-up time, time to leave home in the morning, use of ITNs	Indoor and outdoor HLC, 7:00 p.m.–7:00 a.m.	Mosquito collections: September–October 2009, February–March 2010 Survey: April 2010	Indoor exposure for non-user (*An. funestus)*: 98% Indoor exposure for non-user (*An. quadriannulatus*): 97% Indoor exposure for ITN-user (*An. funestus*): 57% Indoor exposure for ITN-user (*An. quadriannulatus)*: 58%
Huho et al. (2013) [24]	Burkina Faso Kenya Tanzania Zambia	Burkina Faso and Tanzania: Direct observation by field worker 6:00 p.m. until all household members went to sleep and 4 a.m.–6 a.m. Kenya and Zambia: Malaria indicator survey	Observation: Household members awake, by hour Survey: To the nearest hour, time that each household member went indoors, to bed, woke up, and left the home	Indoor and outdoor HLC, start time ranged from 6:00 p.m. to 8:00 p.m. and end time ranged from 6:00 a.m. to 7:00 a.m. across sites	Tanzania and Burkina: 2001 and 2004 Kenya and Zambia: 2009 and 2010 Exact timing of entomological and human behavioural data collection was not provided	Indoor exposure for non-user (*An. gambiae* s.l.): ranged from 87 to 97% across sites Indoor exposure for non-user (*An. funestus* s.l.): ranged from 62 to 97%
Bayoh et al. (2014) [25]	Kenya	Survey	ITN use, usual time indoors for the night, bed time, wake-up time, time to leave home in the morning	Indoor and outdoor HLC, 5:00 p.m.–7:00 a.m.	Mosquito collections: June–July 2011 Survey: July–August 2011 Data compared to data from previous study carried out in 1989–1990	Indoor exposure for non-user: > 90% in all years Indoor exposure for non-user during sleeping hours: ≥ 90% for all species except for *An. arabiensis* (97% in 1989/1990; 80% in 2009; 84% in 2011) Indoor exposure for ITN-user during sleeping hours: (64–77% in 1989–1990; 20–52% in 2009 and 2011)
Moiroux et al. (2014) [26]	Benin	Survey	Time each household member entered and exited the house the night before the survey and entered and exited the sleeping space	Indoor and outdoor HLC, 11:00 p.m.–9:00 a.m.	Mosquito collections: April 2011 Survey: March 2013	Indoor exposure for non-user: 86% and 94% in the two study sites Protective efficacy of an ITN: 80% and 87% Indoor exposure for ITN-user: 55% and 31%
Cooke et al. (2015) [27]	Kenya	Survey completed by head of household on behalf of household members, using digital watch	Time household members entered and exited the house, time to sleep, and use of ITNs	CDC light-traps set next to occupied ITNs, emptied hourly Indoor traps 5:30 p.m.–5:30 a.m.; outdoor traps 5:30 p.m.–10:30 p.m. only	Mosquito collections: June 2011–May 2012 Survey: June 2011–May 2012	Indoor exposure for non-user: 95% (31% before bed and 64% while asleep) Protective efficacy of an ITN:51%
Bradley et al. (2015) [28]	Equatorial Guinea-Bioko Island	Annual malaria indicator survey	Time household members entered the house the night before, any other time spent outside the house between 7:00 p.m. and 6:00 a.m., bed time	Indoor and outdoor HLC, 7:00 p.m.–6:00 a.m.	Mosquito collections: January-October 2009-2013 Survey: 2013	Indoor exposure for non-user: 80%
Kamau et al. (2017) [29]	Kenya	Survey administered to head of household	Time each household member went to sleep and woke up	HLC and CDC light traps; 6:00 am–6:00 pm	Mosquito collections: July and August 2016 Survey: September and October 2016	Indoor exposure for non-user (children < 5): 90%

Studies that integrated human and vector data used estimates of indoor and outdoor vector biting as well as the distribution of people indoors and outdoors for each hour of the night to produce a weighted estimate of exposure occurring indoors and outdoors. This analytical approach was used to quantify human-vector interaction by Killeen et al. in rural Tanzania [20]. In this study, human landing catches were used to assess nightly mosquito biting behaviour before and after widespread coverage of ITNs. The proportion of time people spent indoors and outdoors was estimated based on self-reported survey questions on when household members went to bed and woke up in the morning. These data were combined with hourly indoor and outdoor biting rates to calculate the proportion of bites that occur indoors for unprotected individuals, the proportion of bites that occur during sleeping hours, and the “true protective efficacy of an ITN”, defined as the overall reduction in nightly biting rate for an ITN user compared to a non-user.

Variations of this approach to measuring human-vector interaction were found in nine subsequent publications [21–29]. Mosquito biting behaviour was measured in the majority of studies using human landing catches (HLC) indoors and outdoors on an hourly basis. Cooke et al. used CDC light traps and human baited ITNs [27]. All of the studies focused mosquito collections on the peri-domestic setting, which included inside of dwellings and in the outdoor space directly outside of the dwelling [30]. Mosquito collections were generally carried out indoors and outdoors, from dusk until dawn, with some variation in start and end times across study sites.

The human behavioural variables of interest and approach used to calculate indoor and outdoor components of human-vector interaction were similar across studies. Like the study by Killeen et al., these studies included estimates of time spent indoors and outdoors throughout vector biting hours, however in some cases they used different methods to derive these estimates. For example, Cooke et al. provided digital watches to heads of household and had them fill out surveys on household members’ night time behaviour [27]. Geissbuhler et al. used self-reported survey data, validated by a smaller number of evening observations [21]. Huho et al. looked at human-vector interaction across countries, using different methods for measuring human behaviour in different locations [24]. This included direct observation of night time behaviour from 6:00 p.m. to bedtime and 4:00 a.m. to 6:00 a.m. in selected sites in Tanzania and Burkina Faso, and self-reported survey data from malaria indicator surveys in Zambia and Kenya. The two methods were not compared to one another. Bradley et al. also used data from questions included in a malaria indicator survey [28]. Other studies used self-reported survey data to gather similar information to Killeen et al. The exact list and phraseology of the questions was not included in all publications, but differences in content were identified. Some studies only asked about the time participants went to bed and woke up, while others included additional questions on time participants went inside the house and time they went outside in the morning to more closely approximate when people were outdoors, indoors and awake, and indoors and asleep.

The most common human-vector indicators presented in the studies reviewed included proportion of exposure to malaria vectors occurring indoors for an unprotected individual, exposure to malaria vectors occurring indoors during sleeping hours for an unprotected individual, exposure occurring indoors for an ITN user, and protective efficacy of an ITN. However, these indicators were not uniformly calculated and were not included across all studies integrating human and vector data.

Across settings, a majority of human exposure to malaria vectors for non-users of ITNs was found indoors, largely during sleeping hours. However, variation was observed across settings. In Western Kenya, Cooke et al. found that without the protection of an ITN over 90% of exposure occurred indoors, similar to estimates from Killeen et al. in the Kilombero Valley of Tanzania, Seyoum et al. in South-East Zambia, Bayoh et al. in western Kenya, Moiroux et al. in south Benin, and Huho et al. in six sites in rural Burkina Faso, Kenya, Zambia, and Tanzania [20, 23–27]. However, Cooke et al. found that use of an ITN could prevent only about half of exposure to malaria vectors despite predominantly endophagic primary vectors, likely due to high levels of indoor exposure before sleeping hours [27]. Kamau et al. estimated the fraction of exposure occurring indoors and outdoors for children under five. Overall, the study estimated that 10% of exposure is happening outdoors, primarily during early evening hours between 6:00 p.m. and 9:00 p.m. [29].

Results suggest shifts in both time and place (indoor/outdoor) of exposure across time. Russell et al. found significant changes in indoor human exposure to malaria vectors for both *An. gambiae* s.l. and *An. funestus* as ITN use increased [22]. In 1997, over 90% of exposure occurred indoors for both vector species; by 2009 when ITN access and use had increased, indoor exposure dropped to 79% for *An. gambiae* s.l. and. 45% for *An. funestus* and a higher proportion of exposure outdoors during early evening hours was observed. The protective efficacy of an ITN varied from approximately 50% to over 80% in studies that reported on it, with even lower protective efficacy (38%) reported for specific vector species, namely *An. arabiensis* [20, 21, 26, 27].

### Association between night time location and malaria risk

Seven studies were identified that linked night time location with malaria risk. Four of the six studies specifically looked at whether time spent outdoors at night was associated with an increased risk of malaria infection. A case control study in South-West Kenya by Githinji et al. assessed micro-ecological and human behavioural factors associated with an increased risk of malaria infection. Human behaviour was assessed through a standardized survey [31]. No detail on the content of the human behavioural survey questions was included in the methods section. Results showed participants who spent time outside at night were more likely to be infected with malaria. Spending time outside at night was binary and did not specify length of time or time of the night. The discussion section described ‘*experiences gathered during data collection period*’ that showed community ceremonies such as funerals were commonly carried out at night, leading to an increased exposure to the risk of mosquito bites. However, no description was provided in the study methods about how night time activities were recorded nor was this information included in the results section.

In two studies, Bradley et al. investigated the association between time spent outdoors and malaria infection on Bioko Island, Equatorial Guinea. In a 2012 publication, Bradley analysed data from an annual malaria indicator survey, which includes a question asking whether a child spent time outside between 10 p.m. and 6 a.m. [32]. Children aged two to fourteen were tested for *Plasmodium falciparum* using rapid diagnostic tests (RDT). Only 4% of children were reported to spend time outside during this time and no significant difference in prevalence was observed for children who spent time outside verses those who did not. In a 2015 publication, Bradley et al. conducted a survey to measure the association between time spent outdoors and malaria infection as measured by RDT, in addition to measuring exposure to malaria vectors [28]. Malaria infection was not significantly higher in individuals who reported spending time outside between 7:00 p.m. and 6:00 a.m. the previous night compared to those who did not, in both adults and children. Malaria infection in neither adults nor in children was associated with exposure to outdoor bites, even after adjusting for confounders.

Mwesigwa et al. assessed incidence of *P. falciparum* infection using a cohort study in The Gambia [33]. The study included a household survey that asked about outdoor sleeping among household members. Outdoor sleeping varied by season and was associated with a significantly higher risk of malaria infection.

Hetzel et al. carried out a longitudinal study looking at time spent at *shamba* (farm houses) and incidence of fever in rural Tanzania [34]. The study included a survey to record where household members spent time during the day and night and use of ITNs as well as a treatment-seeking questionnaire recording fever episodes and treatment-seeking behaviour. During weeding and harvesting seasons a large proportion of household members spent days and nights at the farm houses. Fever incidence rates were lower in the *shamba* compared to the village, and 97% of participants reported using a mosquito net the night before. The discussion noted that since *shamba* houses are spread out there is little opportunity for socializing in the evening and, therefore, household members were likely to go to bed early; however, information on night time social activities and average bed times were not reflected in the study methods or results.

Using global positioning system (GPS) data loggers, Searle et al. assessed seasonal movement patterns in rural Southern Zambia [35]. As part of the study, the team assessed time spent away from the household compound during peak biting hours, defined as 7:00 p.m. to 6:00 a.m. for the primary local vector, *An. arabiensis*. On average, participants spent 5.6% of time away from the household compound during peak vector biting hours. Time spent in high or low risk areas, identified by a malaria risk map, depended on the level of risk for the area in which a participant’s compound was located. Participants largely spent time in and close to their household compound, with less frequent longer distance movements. While the study assessed time away from the household compound during peak biting hours, the spatial resolution of the loggers was not sufficient to distinguish time spent indoors and outdoors specifically.

Ototo et al. included the percentage of the population outdoors during times of the night when the highest densities of blood fed vectors were collected in a study in Kenya [36]. Approximately half of the study population was outdoors in the evening between 6:00 p.m. and 8:00 p.m. and in the morning between 6:00 a.m. and 8:00 a.m. In the highland sites, participants reporting going outdoors earlier in the morning compared to the lowland sites, between 4:00 a.m. and 6:00 a.m., largely due to agricultural activities. The results show that no one slept outdoors, even in the hot months. While presented together, the data were not integrated to provide an estimate of exposure to malaria vectors.

### Characterization of night time activities

A total of 10 studies included some description of night time activities occurring during times when local malaria vectors are active (Table 3). These studies identified activities taking place in the peri-domestic setting (inside and directly outside of the home), as well as away from home, throughout the night. This included routine household chores and entertainment occurring in the evening hours before bed, routine livelihood activities that lasted throughout the night such as security and fishing, and large-scale socio-cultural events, such as weddings and funerals which lasted throughout the night. Circumstances that could temporarily disrupt usual sleeping patterns were also described including travel, illness, and house guests, as well as seasonal changes to sleeping patterns associated with farming practices and outdoor sleeping.

**Table 3:** Studies including description of night time human activities

Author (year) [reference]	Location	Methods used to capture night time activities	Night time activities identified	Night time activity categories
Alaii et al. (2003) [37]	Kenya	Early morning (4:00 a.m.–6:00 a.m.) observation of ITN use Survey question on barriers to ITN use for children under 5	Funeral ceremonies Disruption of sleeping patterns due to visitors	Large-scale socio-cultural events travel/visitors
Dunn et al. (2010) [38]	Tanzania	In-depth interviews Focus group discussions Participatory methods	Spending night at farming plot Outdoor sleeping Socio-cultural events e.g. funerals Household chores (women) Drinking, watching television, and socializing (men)	Livelihood activities Large-scale socio-cultural events Routine household activities Entertainment
Tuno et al. (2010) [30]	Ghana	Survey	Outdoor sleeping	Outdoor sleeping
Monroe et al. (2014) [39]	Uganda	In-depth interviews Focus group discussions	Funerals, weddings, religious events, parties Socializing, visiting bars Overnight visits with friends/family Occupations such as police and fishing Outdoor sleeping Domestic disputes, insurgency, illness	Large-scale socio-cultural events Entertainment Travel/visitors Livelihood activities Outdoor sleeping Times of difficulty
Dlamini et al. (2015) [40]	Swaziland	In-depth interviews Focus group discussion Direct observation from morning to late evening	Soccer playing and socializing (adolescent boys) Drinking at local bars (men) Preparing meals and fetching water (women and adolescent girls)	Entertainment Routine household activities
Monroe et al. (2015) [41]	Ghana	Direct observation (6:00 p.m.–6:00 a.m.) In-depth interviews	Household chores Socializing Weddings, funerals Outdoor sleeping	Routine household chores Entertainment Large-scale socio-cultural events Outdoor sleeping
Swai et al. (2016) [42]	Tanzania	Direct observation (6:00 p.m.–7:00 a.m.) In-depth interviews Focus group discussions	Farming practices Relaxing and storytelling, playing Cooking, eating, fetching water and firewood	Livelihood activities Routine household activities
Moshi et al. (2017) [43]	Tanzania	In-depth interviews Focus group discussions	Cooking, eating, household chores Socializing, drinking at bars	Routine household activities Entertainment
Masalu et al. (2017) [44]	Tanzania	Focus group discussions	Farming, night guard, sex work Funerals, parties, and gatherings	Livelihood activities Large-scale socio-cultural events
Makungu et al. (2017) [45]	Tanzania	In-depth interviews Focus group discussions Photovoice methods	Household chores Watching television, drinking at bars Fishing, street vending Funeral ceremonies	Routine household activities Entertainment Livelihood activities Large-scale socio-cultural events

Methods used to document and characterize human behaviour included in-depth interviews, focus group discussions, participatory methods (e.g. mapping, diagramming, and photovoice recordings), direct observation of night time events, and questionnaires. These studies often looked at specific night time activities, as well as the impact of these activities on use of malaria prevention tools.

The level of detail provided on night time activities and sleeping patterns varied widely across publications. In some studies, night time human behaviour was the primary area of focus. For example, Dunn et al. explored shifting household sleeping patterns in response to livelihood practices and socio-cultural events, and how these could impact malaria exposure and prevention practices in rural Tanzania [38]. The study documented changes in daily and seasonal sleeping patterns associated with farming practices that could impact human-vector interaction either through differences in time spent indoors/outdoors or differences in use of ITNs. The study also identified risk behaviours during socio-cultural events, such as funeral ceremonies, that could impact time spent outdoors and use of ITNs.

In a study by Monroe et al. in Uganda, spending time away from home at night emerged as an important theme for understanding potential malaria exposure and prevention practices [39]. Social events, livelihood activities, and "times of difficulty” were identified as circumstances in which people spend part or all of the night away from home. Social events included funerals, weddings, religious ceremonies, spending time at bars and discos, and visiting friends and family. Livelihood activities included professions such as police, security guards, soldiers, fishermen, and brick-makers who might stay outside all night as part of their job. Times of difficulty at the family and community level included domestic violence or disputes, and security issues. Social barriers inhibited net use away from home as people feared being perceived as rude or as showing off if they brought their nets to large or small-scale social events. Not having a place to hang the net, or not having enough nets at home to take one when staying away, were also barriers.

A study by Monroe et al. in northern Ghana included both in-depth characterization of night time activities and assessment of potential human-vector interaction. In addition to in-depth interviews with community members and health workers, and semi-structured observations of night time activities and sleeping patterns throughout the night, the study team observed when people were indoors or outdoors, under a net, and sleeping for each household member at half-hourly intervals throughout the night. Entomology data were not collected as part of the study, however biting times from entomological monitoring in nearby sites was discussed in relation to human activities and sleeping patterns throughout the night [41]. This study identified a range of routine household chores, social activities, and large-scale events that may impact exposure to malaria vectors and use of prevention tools. Large-scale socio-cultural events and outdoor sleeping were the most common reasons for people to be outside during peak vector biting hours in the middle of the night.

In other studies, night time activities were included to a smaller extent as part of a larger research study. Alaii et al. monitored ITN use through early morning observations following net distribution. A household survey included a question on reasons why children under five might not use a net. Among those that provided reasons, social reasons accounted for a third of responses and included disruption of sleeping arrangements, funerals, visitors, and illness. Entomological indices were calculated in control villages but were not integrated with the human behavioural data [37].

Masalu et al. conducted a study to test transfluthrin-treated decorative baskets and wall decorations at bars and included a small number of focus groups to assess acceptability of the products [44]. Focus group discussion respondents noted common night time activities, including farming, night guarding, sex work, funerals, parties, and other gatherings as activities that could increase risk of exposure to malaria. Mosquito collections were carried out in bars but were not linked with human behavioural data.

Dlamini et al. used a combination of semi-structured interviews, focus group discussions, and observations to identify behaviours that might impact malaria control interventions. Group socialization outside late into the evening at soccer games, friends’ houses, or drinking establishments was found to be the primary behaviour keeping people from using ITNs during vector biting hours. These activities were most common among young men. Preparing meals and fetching water were identified as common activities for women and girls [40].

A study by Tuno et al. in Ghana included a survey to determine the frequency of outdoor sleeping in study sites as well as where and when participants slept outdoors. The findings from this component of the study were presented separately from the entomological component. A significant proportion of men (37% and 82%) and women (16% and 56%) reported sleeping outdoors for a portion of the night in the two sites.

Swai et al. looked at biting risk associated with migratory farming practices. In addition to looking at mosquito biting behaviour, the frequency of night time human activities (cooking, eating, washing dishes, fetching water and firewood, and storytelling) was recorded through direct observation. These activities were frequently observed during the times when the highest biting rates were recorded for local vectors, between 6:00 p.m. and 11:00 p.m. While human and vector data were collected and analysed together to describe where humans may be at risk, the data was not integrated to provide a quantitative estimate of indoor and outdoor exposure based on the distribution of humans and vectors throughout the night [42].

As part of a qualitative study, Moshi et al. used in-depth interviews and focus group discussions to better understand community knowledge of malaria transmission. The study described time spent outdoors, primarily during the early evening hours. During this time household chores, such as cooking, and socializing were common. Sitting outdoors at bars was described as a common activity for males [43]. Likewise, Makungu et al. used focus group discussions, in-depth interviews, and photovoice methods to capture perceptions and practices around mosquito control. Participants described gaps in protection when they were outdoors, particularly during livelihood and leisure activities. Examples of activities that kept people outside at night included fishing, street vending, watching television, drinking at bars, and attending funeral ceremonies [45].

While some differences were noted across settings, activity categories were largely consistent across studies, including activity timing, duration, frequency, and location (Table 4). Within the peri-domestic space, household members engaged in chores, socializing, and relaxing on a nightly basis. Likewise, entertainment activities and small business activities occurred within the community, away from the peri-domestic setting, on a nightly basis, most commonly for adolescent and adult males. Livelihood activities occurred nightly or seasonally and impacted a smaller segment of the study populations. Large-scale social events such as weddings, funerals, and religious events were common across settings and often involved males and females of all ages.

**Table 4:** Night time Activity Categories

Activity category	Population	Frequency	Timing	Location
Routine household activities	Common across settings; involves a large segment of the population; household chores most common among adolescent and adult females	Daily	Evening and early morning	Indoors and outdoors within the peri-domestic space
Routine livelihood activities e.g. security	Common across settings; most common among adult males	Daily	All-night	Outdoors within the community or beyond
Seasonal livelihood activities e.g. farming	Varies by setting	Seasonal	Early morning and evening or in some cases staying at farm plots for days or weeks	Away from home
Large-scale socio-cultural events	Common across settings and involves a large segment of the population	Variable	All-night	Outdoors within the community or beyond
Entertainment e.g. bars, watching television	Common across settings; most common among adolescent and adult males	Daily	Evening and late night	Outdoors within the community
Travel/visiting	Varies by setting	Variable	All-night	Outside of the community; likely indoors
Outdoor sleeping	Varies by setting	Seasonal	Part or all of the night	Near the home, in open air spaces

## Discussion

This review identified two categories of importance related to night time human behaviour. The first relates to when (time of night) and where (indoors versus outdoors) people are exposed to malaria vectors. The second is *what* people are doing at night that may increase their contact with malaria vectors. This understanding of human behaviour is crucial for targeting context-appropriate vector control interventions across settings.

While it was not possible to compare study results directly due to differences in study design and methods, the results of the studies in this review suggest a majority of exposure to malaria vectors continues to occur indoors during sleeping hours for unprotected individuals. This is true even in contexts where unweighted biting rates are higher outdoors than indoors. However, when looking at ITN users, roughly half of exposure occurred outdoors in some settings, signalling a gap in protection. One of the most relevant indicators for understanding residual malaria transmission is the protective efficacy of ITNs, defined as the proportion of human exposure to malaria vectors prevented by ITN use out of total exposure i.e. compared to a non-user. Protective efficacy, the overall reduction in nightly biting rate for an ITN user compared to a non-user, was as low as 50% in some settings, with even lower estimates of protection for primarily exophagic malaria vectors. The fraction of exposure occurring indoors during non-sleeping hours and outdoors can pose a threat to malaria control and elimination efforts [10].

While the review focused on studies published between 2000 and 2017, the importance of considering both vector and human behaviour was put forward as early as 1964. First referred to as “man-biting rate” in a World Health Organization publication, Garrett-Jones described measurement of contact between humans and mosquito vectors, including examples of its use in Mexico and Zanzibar [46]. The “man-biting rate” comprised indoor and outdoor components of mosquito contact. Garrett-Jones highlighted the importance of considering not only mosquito biting rates, but also where humans stay during biting times. He explained that humans as well as mosquitoes must be studied, including their distribution throughout the night [46]. Nearly half a century later a commentary by Linblade emphasized that an understanding of human behaviour is as important as vector behaviour for understanding when and where malaria transmission occurs and that the presence of humans must be considered when calculating risk of infective bites [47].

Despite the importance of human behaviour to understanding malaria transmission dynamics, relatively few studies were identified that included it. Further, differences in methodological approaches were identified across studies, limiting the comparisons that could be made. Moreover, estimates of exposure away from the peri-domestic setting are lacking. Analytical approaches measuring human-vector interaction should account for outdoor sleeping as well as segments of the population that may spend most or all of the night away from home. When possible, human and vector data should be collected close in time and location, and across time points, to reflect changes in vector and or human behaviour across seasons and over time.

A standardized approach and further validation of the estimates provided by different methodologies for collecting human behavioural data will be important next steps. Once validated, a small set of survey questions with uniform phraseology would allow for comparison of human exposure to malaria vectors across settings and over time on a large scale, as well as the evaluation of vector control tools. At a minimum, the human behavioural component should include estimates of the proportion of the population indoors/outdoors throughout the night. This information can be integrated with indoor and outdoor biting rates to calculate a weighted estimate of human exposure to malaria vectors indoors and outdoors. Information on the proportion of the population under an ITN and sleeping during times when malaria vectors are active can provide higher-resolution information on exposure by accounting for ITN use. These data can be used to quantify human exposure to malaria vectors occurring indoors and outdoors, exposure prevented by current ITN use practices, potential gains that could be made through optimizing ITN use during sleeping hours, and exposure that can only be prevented by supplemental tools.

Beyond understanding when and where exposure is occurring, it is crucial to characterize night time activities and sleeping patterns that can put people at risk. The results of the review suggest there are broad night time human activity categories that may be similar across settings in sub-Saharan Africa, including household chores, entertainment, livelihood events, and large-scale community events. Occurrence of outdoor sleeping varied across settings and could be an important factor to consider in settings where the practice is common.

In the context of high access to and use of ITNs, it will become increasingly important to understand gaps in protection. Local information is needed to identify the relative importance of activity categories and target groups based on the entomological, human behavioural, and epidemiological context. The activity categories identified in this review provide a useful framework for informing context-specific research on the relative importance of these activities that can drive locally appropriate interventions.

There are a number of limitations associated with this review. It is possible that studies that would have met inclusion criteria were not identified in the review process. This review did not cover factors that could influence transmission dynamics such as large-scale population movements and internally displaced populations. While important topics to consider, they were outside the scope of this review. However, by not focusing on population movement in the review it is possible that relevant articles could have been missed. Nonetheless, a comprehensive and structured process was utilized. Additionally, inclusion and exclusion criteria and search strategy were determined a priori, thus limiting potential bias in article selection. Lack of standardization in methods across studies precluded meta-analysis and underscored the potential gains to be made from a standardized approach to future collection of these types of data.

## Conclusions

Where possible, studies should include human behavioural research to better understand night time activities and sleeping patterns as they relate to malaria risk. Moving forward, entomological studies should include parallel human behavioural research. A standardized approach will enable tracking of human-vector interaction and gaps in protection provided by ITNs, and other vector control interventions, over time and across settings. This information is essential for strategic targeting of existing tools, effective social and behaviour change interventions, and development and deployment of appropriate complementary prevention tools.

## Abbreviations

ACT: artemisinin-based combination therapy
GPS: global positioning system
HLC: human landing catch
IPTp: intermittent preventive therapy during pregnancy
IRS: indoor residual spraying
ITN: insecticide-treated net
RDT: rapid diagnostic test
WHO: World Health Organization
